# An Evil Which Must Be Ended

**Published:** 1919-01-04

**Authors:** 


### THE EDITOR'S LETTER-BOX.

**[Correspondence on all subjects is invited, but we cannot in any way be responsible for the opinions expressed by our correspondents, who must give their name and address as a guarantee of good faith, but not necessarily for publication. Correspondents are reminded that brevity of style and conciseness of statement greatly facilitate early insertion.]**

### AN EVIL WHICH MUST BE ENDED.

### NIGHT DUTY TORMENTS FOR PATIENTS.

*To the Editor of* The Hospital.

Sir,—I, too, am amongst those wishing to see, at long length, the present common system of hospital night nursing get the full examination it deserves. (I say the *common* system because there must be some exceptions.) There are not many who have been either nurses or patients in hospital who could not say something in strong condemnation of this system if they chose, but from nurses only perhaps can we hope to see pronouncements by the medium of The Hospital.

But if these do not embrace the opportunity for safe "articulation" so freely permitted them there it can only be for reasons that have stifled criticism so long. Outspokenness on the part of the very young nurse—the probationer (who almost always, next to the patient, is the worst sufferer under the system)— is hardly possible, while older nurses have either left hospital and are only too ready to forget its hardships; or, having stayed on, have attained to emancipation and all the worries thereof. These, too, seem often to forget, or worse—remember only in their own interests, to perpetuate upon others the trials they once so much deplored. If nurses *would* but speak out, and you could allow space for it, I fancy they would surprise even one another with the things that different ones have known included in night duty.

"Looking Backwards."

*To the Editor of* The Hospital.

